# LIMITATIONS OF POLYGENIC RISK SCORES FOR DEMENTIA IN FAMILIAL EXCEPTIONAL AGING: IMPACT OF SAMPLING

**DOI:** 10.1093/geroni/igae098.0028

**Published:** 2024-12-31

**Authors:** Laura Xicota, Rong Cheng, Sandra Barral Rodriguez, Lawrence Honig, Joseph Lee

**Affiliations:** Columbia University Irving Medical Center, New York, New York, United States; Columbia University, New York, New York, United States; Columbia University, New York, New York, United States; Columbia University Irving Medical Center, New York, New York, United States; Columbia University, New York, New York, United States

## Abstract

Genetic risk factors for late-onset Alzheimer’s disease (LOAD) have been extensively studied in the general population of European background. Indeed, significant genetic variants with low to moderate contributions to LOAD have been used to calculate Polygenic Risk Scores (PRS) to identify individuals with a higher genetic risk of LOAD. The Long Life Family Study (LLFS) is characterized by having exceptionally long-lived individuals at lower risk of LOAD. With this study, we will uncover whether the described genetic risk factors for LOAD impact AD risk in LLFS, limited to US sites. In LLFS, PRS based on two large GWASs in the general population did not predict clinical AD status. The LLFS population did not have lower allele frequencies of risk alleles, but rather lacked putative effects in these risk alleles, with only 6.7% of SNPs from GWAS 1 and 7.7% from GWAS 2 were associated with AD. We then examine the relations between PRS and endophenotype, namely AD biomarkers (Aβ40, Aβ42, Aβ42/Aβ40, NfL, and GFAP). Levels of AD biomarkers were not positively associated with PRS, which can be explained by the low frequencies of SNPs associated with biomarker concentrations (3.3%-6.7% for GWAS 1; 2.6%-6.5% for GWAS 2). Here, we show that genetic risk factors for AD in the general population do not necessarily explain AD risk in a cohort of long-lived individuals; therefore, genetic risk factors based on large-scale GWASs ascertained from the general population need to be examined with caution when applied to a cohort ascertained for exceptional longevity.

